# Chemotaxonomic Insights into Korean *Daphne* spp. and *Wikstroemia* spp. by Integrating Flavonoid Contents with Ecological Factors

**DOI:** 10.3390/plants14193059

**Published:** 2025-10-03

**Authors:** Yonghwan Son, Ji Ah Kim, Ho Jun Son, Hyun-Jun Kim, Wan-Geun Park

**Affiliations:** 1 Forest Medicinal Resources Research Center, National Institute of Forest Science, Yeongju-si 36040, Republic of Korea; thsdydghks@korea.kr (Y.S.); jiahkim@korea.kr (J.A.K.); shj7400@korea.kr (H.J.S.); 2 Department of Forest Resources, College of Forest and Environmental Sciences, Kangwon National University, Chuncheon-si 24341, Republic of Korea

**Keywords:** chemotaxonomic study, *Daphne*, flavonoids, Thymelaeaceae, *Wikstroemia*

## Abstract

The placement of *Daphne genkwa* has long been controversial, as its intermediate morphological traits blur the boundary between *Daphne* and *Wikstroemia*. To address this challenge, we adopted a chemotaxonomic approach, integrating flavonoid contents with ecological indicators, as an independent line of evidence complementing morphology and molecular data. Using UPLC-UV, six flavonoids were quantified from 16 Korean populations representing six taxa. Multivariate analyses clearly distinguished *Daphne* and *Wikstroemia*, with *D. genkwa* and *W. ganpi* forming closely related but separate clades. Ecological factors such as precipitation and canopy openness significantly affected flavonoid levels, particularly luteolin 7-*O*-glucoside and yuankanin. However, the diagnostic flavonoid fingerprints remained consistent across habitats. This study demonstrates that integrating chemical and environmental perspectives can strengthen taxonomic frameworks and support both classification and chemotaxonomic evidence in Thymelaeaceae, offering a methodological basis for future comparative studies across related plant families.

## 1. Introduction

The family Thymelaeaceae, first circumscribed in Genera Plantarum by Jussieu [1], has been progressively refined through morphological syntheses and today comprises about 48 genera and 650 species distributed from temperate to tropical regions worldwide [2,3,4]. In East Asia it occurs in China, Japan, Korea, and parts of Russia, with five genera and ten species recognized in Korea [5]. Nevertheless, the distinction between *Daphne* and *Wikstroemia* is still debated, because *Daphne genkwa* frequently displays intermediate traits of both genera, a pattern that has been repeatedly noted in previous studies [6,7,8]. Recent plastid genome sequencing and DNA barcoding have consistently placed *D. genkwa* within *Wikstroemia* and confirmed a sister group relationship between the two genera [9,10]. However, both *D. genkwa* and *Wikstroemia genkwa* continue to be used in major references, reflecting incomplete nomenclatural consensus [11,12,13,14]. This situation highlights the value of chemotaxonomic research as an advanced and integrative framework for taxonomy [15]. Complementing morphological and genetic studies, it offers a deeper perspective that connects molecular systematics with ecological factors [16].

Chemotaxonomy, defined as the systematic use of secondary metabolites in classification, has long been recognized as a valuable complement to morphology and molecular biology [17,18]. Flavonoids in particular are among the most widely applied chemotaxonomic indicators because of their structural diversity, widespread distribution, and relative stability compared with other specialized metabolites [19,20]. Their presence or absence and relative abundance often align with taxonomic boundaries, providing lineage- specific indicators that help resolve ambiguities when morphological characters overlap [21,22]. Such approaches have also been successfully applied in other plant groups, including *Sideritis* [23], *Salvia* [24] and Campanulaceae species [25], where secondary metabolite patterns provided reliable evidence for resolving taxonomic relationships. Beyond their systematic value, flavonoids also reflect ecological interactions and adaptive responses, further supporting their reliability as taxonomic indicators [26,27,28,29]. Thus, flavonoid-based chemotaxonomy offers robust and independent evidence that complements plastid genome sequencing and DNA barcoding, thereby providing a more comprehensive framework for refining classification within Thymelaeaceae.

Despite its considerable strengths, chemotaxonomy also has inherent limitations [16]. Because secondary metabolites are not fixed traits but dynamic products of plant physiology, their expression and relative abundance can be influenced by developmental stage, organ specificity, and physiological status [30]. Numerous studies have shown that environmental conditions influence the biosynthesis and accumulation of specialized metabolites [31,32]. Consequently, chemical profiles may indicate not only taxonomic boundaries but also ecological adaptation. This dual nature is both a challenge and an opportunity [16]. If environmental influences are ignored, chemotaxonomic evidence may be misinterpreted or oversimplified [33]. However, by explicitly incorporating ecological variables such as temperature, precipitation, altitude, soil chemistry, and light availability, chemotaxonomic research can move beyond static chemical fingerprints and achieve a more dynamic interpretation [32,34,35]. In this expanded framework, metabolite profiles serve not only as markers for lineage delimitation but also as windows into the ecological strategies and adaptive potential of taxa [30,33].

Building on this framework, we applied a chemotaxonomic approach to Korean Thymelaeaceae, integrating flavonoid profiles with ecological, morphological, and molecular evidence. *Daphne genkwa* has long been recognized as an ambiguous taxon, yet it is also a traditional medicinal plant that has been extensively investigated pharmacologically, with numerous flavonoids and related metabolites already identified [11,12,35,36,37,38,39]. Because these flavonoids are well documented as major constituents in the leaves and aerial parts, we employed them for UPLC-UV profiling of six major compounds: luteolin 7-*O*-glucoside (L7OG), yuankanin, apigenin, luteolin, hydroxygenkwanin, and genkwanin [40,41,42,43]. Notably, yuankanin is a 5-diglycoside derivative of genkwanin, and genkwanin itself is a methylated form of apigenin, while hydroxygenkwanin represents another closely related methoxylated flavone. These structural relationships emphasize the biosynthetic links among the quantified compounds and provide a clear foundation for interpreting the correlations observed between apigenin, genkwanin, hydroxygenkwanin, and yuankanin in the subsequent analyses. These taxa are also listed in the National Red List, with categories ranging from Endangered (EN) to Least Concern (LC), highlighting their conservation relevance [44,45]. This approach allowed us to evaluate whether flavonoid composition retains diagnostic value at the genus and species levels while also capturing ecological responses to environmental heterogeneity.

## 2. Results

### 2.1. Sampling Sites and Environmental Conditions

Across the 16 populations, a wide range of geographic, climatic, and habitat variables was observed (Table 1 and Table S1). Sites ranged from 33.32° N (*D. jejudoensis*, Jeju Island) to 37.72° N (*W. trichotoma*, nearshore island) and from 126.28° E to 129.41° E. Elevations varied widely, from 33.2 m (*W. genkwa*, coastal lowland) to 1192 m (*D. pseudomezereum* var. *koreana*, subalpine mountain), with a median around 128 m. Average annual temperature (BIO1) spanned 11.8–16.9 °C, with the highest values at *D. jejudoensis* sites and the lowest at *D. pseudomezereum* var. *koreana*. Maximum temperature of the warmest month (BIO5) reached up to 28.0 °C in southern coastal and island sites, while minimum temperature of the coldest month (BIO6) dropped below −11.3 °C in subalpine mountain populations. Annual precipitation (BIO12) ranged from 1170 mm (*W. genkwa*) to 1770 mm (*D. jejudoensis*), with southern coastal populations (*W. ganpi*, *W. genkwa*) generally receiving higher rainfall. Habitat structure also differed considerably, with slope ranging from 3° (*W. ganpi*) to 60° (steep inland *W. ganpi* sites), and canopy cover index (CCI) from completely open (0%, *W. genkwa*) to dense canopy (88%, *D. kiusiana*).

### 2.2. Method Validation and Quantitative/Qualitative Analysis of Six Flavonoids

Six flavonoids (L7OG, yuankanin, luteolin, apigenin, genkwanin, and hydroxygenkwanin) were simultaneously quantified within 40 min using a gradient elution system with acetonitrile and distilled water. The developed method showed excellent linearity (R^2^ > 0.999) over wide calibration ranges for all compounds (Table 2). Limits of detection (LOD) and quantification (LOQ) were low, ranging from 0.01 to 0.06 μg/mL and 0.02–0.19 μg/mL, respectively. Precision was high, with intra- and inter-day relative standard deviations (RSD) below 1.1%, while accuracy values ranged from 95.9% to 104.3% (Table 3). These results demonstrate that the method is reliable and reproducible for the quantification of the six flavonoids.

Qualitative analysis revealed clear chemotaxonomic patterns among the six species (Table S2). *W. genkwa* and *W. ganpi* shared three compounds (L7OG, genkwanin, and hydroxygenkwanin) but were distinguished by the exclusive presence of yuankanin in *W. genkwa*. In contrast, only apigenin was detected in *W. trichotoma*. Within the genus *Daphne*, five compounds were identified: L7OG, luteolin, apigenin, hydroxygenkwanin, and genkwanin, with luteolin present in all three examined species. *D. pseudomezereum* var. *koreana* was characterized by the presence of apigenin and genkwanin, while *D. jejudoensis* and *D. kiusiana* were distinguished by hydroxygenkwanin.

Quantitative analyses (expressed as mean ± SD, μg/mL, based on three independent replicates) supported these qualitative distinctions. In *W. genkwa*, L7OG (766.94 ± 191.72), yuankanin (562.64 ± 76.90), and hydroxygenkwanin (343.48 ± 108.23) were the predominant constituents, along with moderate levels of genkwanin (28.48 ± 8.27). *W. ganpi* exhibited high concentrations of apigenin (32.60 ± 6.45) and hydroxygenkwanin (432.72 ± 122.08), together with L7OG (16.61 ± 3.46) and genkwanin (17.05 ± 3.86). In *W. trichotoma*, apigenin (3.57 ± 0.24) was the only detected compound. In the *Daphne* group, *D. pseudomezereum* var. *koreana* accumulated luteolin (5.97 ± 0.81) and apigenin (3.10 ± 0.69), whereas *D. jejudoensis* was characterized by luteolin (6.61 ± 0.91) and L7OG (10.13 ± 1.57). *D. kiusiana* contained relatively high amounts of L7OG (29.37 ± 9.38), luteolin (10.79 ± 3.32), and hydroxygenkwanin (2.18 ± 0.65).

### 2.3. Correlation Between Environmental Variables and Flavonoid Contents

**Figure 1:**
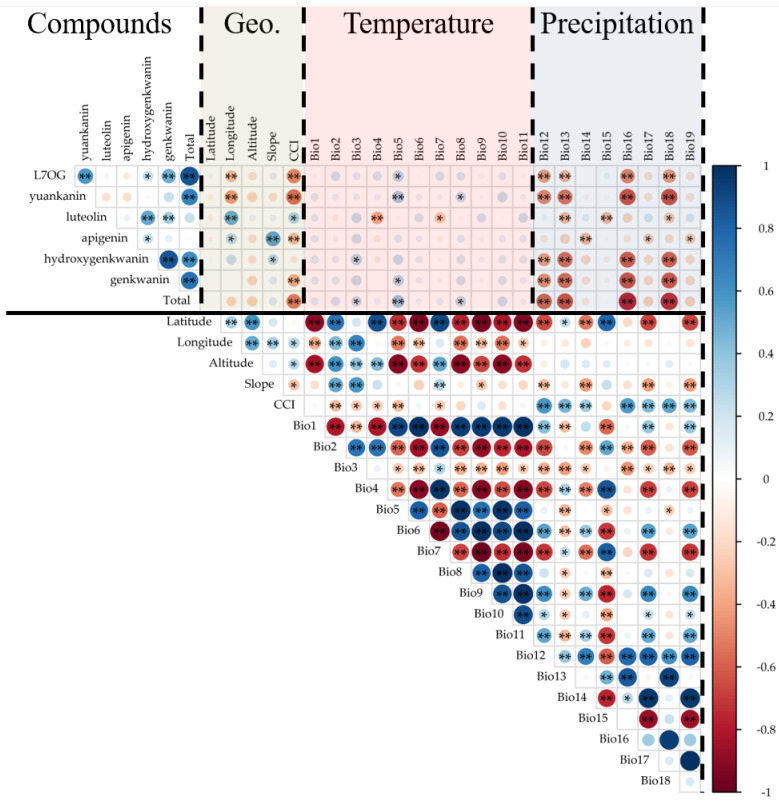
Correlation matrix between compounds and environmental variables (Geo., temperature, and precipitation) in Korean Thymelaeaceae. Circles represent Pearson’s correlation coefficients, with size and color indicating strength and direction (blue = positive, red = negative). Significance levels: * *p* < 0.05; ** *p* < 0.01.

Correlation analysis between the flavonoid contents and environmental variables was performed (Tables S3 and S4 and Figure 1). Among the geographic and topographic factors, longitude was negatively correlated with L7OG (*r* = −0.311, *p* < 0.01) and yuankanin (*r* = −0.421, *p* < 0.01), but positively correlated with luteolin (*r* = 0.482, *p* < 0.01) and apigenin (*r* = 0.267, *p* < 0.05). Altitude showed a significant positive association with apigenin (*r* = 0.527, *p* < 0.01) but was negatively correlated with L7OG (*r* = −0.488, *p* < 0.01). Similarly, CCI was negatively related to L7OG (*r* = −0.488, *p* < 0.01), yuankanin (*r* = −0.558, *p* < 0.01), and genkwanin (*r* = −0.369, *p* < 0.01).

Regarding the bioclimatic variables, temperature-related factors (BIO1–BIO11) showed complex associations with flavonoid contents. For example, Bio4 was negatively correlated with luteolin (*r* = −0.356, *p* < 0.01), while Bio5 showed positive correlations with L7OG (r = 0.272, *p* < 0.05) and yuankanin (*r* = 0.338, *p* < 0.01). Precipitation-related factors (BIO12–BIO19) revealed consistent patterns, with Bio16 negatively associated with L7OG (r = −0.489, *p* < 0.01) and yuankanin (*r* = −0.637, *p* < 0.01), and BIO18 negatively correlated with hydroxygenkwanin (*r* = −0.587, *p* < 0.01) and genkwanin (*r* = −0.613, *p* < 0.01).

Overall, these results indicate that both geographic/topographic and climatic variables influenced flavonoid accumulation, with temperature and precipitation exerting particularly strong effects. In addition, the sum of total flavonoid content also showed significant associations with environmental variables. Specifically, total flavonoids were negatively correlated with canopy cover index (*r* = –0.516, *p* < 0.001) and precipitation-related factors, including BIO12 (*r* = –0.403, *p* < 0.01), BIO16 (*r* = –0.648, *p* < 0.001), and BIO18 (*r* = –0.484, *p* < 0.01). These results suggest that overall flavonoid accumulation provides a clearer ecological signal than individual compound responses.

### 2.4. Multivariate Analysis of Flavonoid Profiles

**Figure 2:**
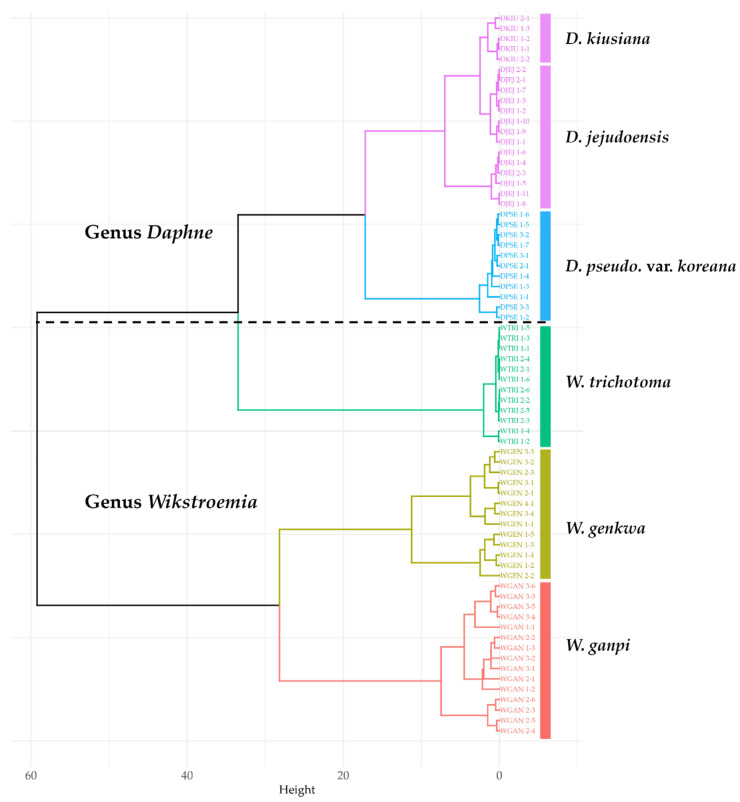
Hierarchical clustering of six Thymelaeaceae taxa based on flavonoid profiles. WGAN: *Wikstroemia ganpi*; WGEN: *W. genkwa*; WTRI: *W. trichotoma*; DPSE: *D. pseudomezereum* var. *koreana*; DJEJ: *D. jejudoensis*; DKIU: *D. kiusiana*.

Qualitative and quantitative data for six flavonoids in six taxa were used for HCA (Figure 2). The results revealed two major clades and five subclades. Clade 1 was divided into two subclades, separating *W. genkwa* and *W. ganpi* as distinct but closely related groups. Clade 2 consisted of three subclades: *W. trichotoma* formed an independent cluster, *D. pseudomezereum* var. *koreana* constituted a separate subclade, and *D. kiusiana* and *D. jejudoensis* clustered closely together, indicating a strong relationship between the two species.

**Figure 3:**
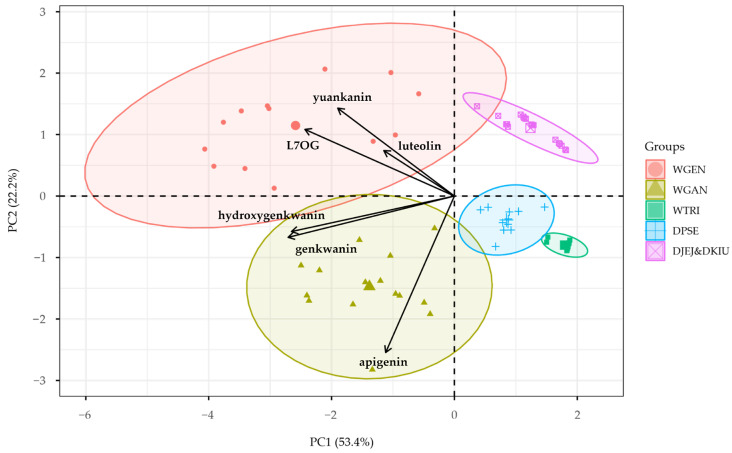
Principal component analysis (PC1&PC2) of Thymelaeaceae taxa based on flavonoid profiles. WGAN: *Wikstroemia ganpi*; WGEN: *W. genkwa*; WTRI: *W. trichotoma*; DPSE: *D. pseudomezereum* var. *koreana*; DJEJ: *D. jejudoensis*; DKIU: *D. kiusiana*.

**Figure 4:**
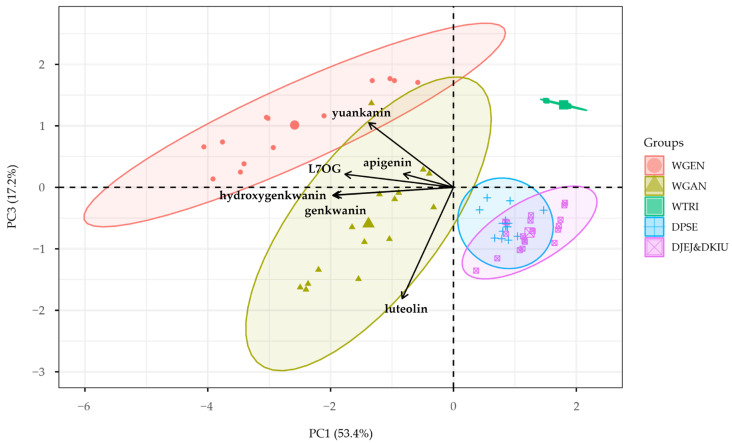
Principal component analysis (PC1&PC3) of Thymelaeaceae taxa based on flavonoid profiles. WGAN: *Wikstroemia ganpi*; WGEN: *W. genkwa*; WTRI: *W. trichotoma*; DPSE: *D. pseudomezereum* var. *koreana*; DJEJ: *D. jejudoensis*; DKIU: *D. kiusiana*.

Subsequently, PCA was performed based on the HCA groupings (Figure 3 and Figure 4). The first principal component (PC1) explained 53.4% of the total variance, with hydroxygenkwanin and genkwanin as the main contributors. The second component (PC2) accounted for 22.2% of the variance, driven mainly by apigenin, and the third component (PC3) explained 17.2% of the variance, with luteolin as the primary variable. PC1, with an eigenvalue of 3.2025, was identified as the most significant axis, and the cumulative variance explained by the first three components was 92.9%. The PC1&PC2 plot showed a clear separation between *W. genkwa* and *W. ganpi*, distinguished by hydroxygenkwanin and genkwanin, while the PC1&PC3 plot indicated that *W. trichotoma* formed an independent cluster, with the three *Daphne* taxa grouping together.

## 3. Discussion

In this study, UPLC-UV-based quantitative and qualitative analyses were used to elucidate the interactions between environmental factors and flavonoid metabolites in Korean Thymelaeaceae, while confirming that chemotaxonomic structure at the genus and species levels is retained. In particular, among the six flavonoids, *Wikstroemia* species showed elevated hydroxygenkwanin and genkwanin contents, and these differences consistently separated taxa at genus and species levels in multivariate analyses.

Flavonoids are multifunctional metabolites that respond dynamically to environmental variables, contributing to plant tolerance against both abiotic and biotic stresses [30,31]. Because of this ecological plasticity, environmental heterogeneity among habitats is widely recognized to influence flavonoid profiles [46,47]. For instance, variation in total flavonoid content has been reported in *Plumula nelumbinis* [48], while compositional differences in kaempferol and quercetin have been observed in *Lathyrus japonicus* [49]. Consistent with these observations, our study revealed pronounced regional differences in L7OG, yuankanin, and hydroxygenkwanin in *W. genkwa*.

Such chemical divergence reflects the broad ecological gradient across these taxa. The six taxa examined occupy habitats ranging from the cool, humid subalpine zones of *D. pseudomezereum* var. *koreana* to the warm, dry coastal environments of *W. genkwa* [44,45]. These contrasting habitats likely contribute to the differences in cumulative flavonoid yields observed between the two genera. In the genus *Daphne*, adapted to more shaded conditions, tended to show comparatively lower levels, whereas *Wikstroemia* taxa from more exposed slopes exhibited higher accumulations. Such patterns may also be linked to micromorphological traits, including epidermal thickness and trichome density, which modulate light interception and water retention and thereby influence flavonoid investment [6,44]. Correlation analyses also revealed that environmental context strongly influences flavonoid composition, with canopy openness and water availability emerging as the most consistent ecological drivers. Although Pearson’s correlation coefficients were relatively low, such modest values are expected because flavonoid accumulation is influenced by multiple environmental and physiological factors, which often weaken simple linear relationships [50,51]. Importantly, when considering total flavonoid content, the correlations with CCI and precipitation indices became more consistent, indicating that cumulative flavonoid production provides a clearer ecological signal than individual compounds. Flavones function as protective metabolites under UV-B exposure and water limitation, enabling plants to cope with light and drought stress [52]. For example, in maize, UV-B treatment significantly increased apigenin accumulation by activating the flavone synthase I/II pathway [53]. More broadly, exposure to excess light or UV-B stimulates the synthesis of dihydroxy B-ring-substituted flavonoids, such as luteolin derivatives [54]. In line with these mechanisms, our results showed that CCI was negatively correlated with flavonoid contents, indicating that plants in open habitats upregulate flavonoid biosynthesis as an adaptive response to higher light stress. Similarly, water-related variables (BIO12-19) displayed overall negative correlations with flavonoid levels, consistent with previous studies reporting that drought stress enhances the expression of flavonoid biosynthetic genes (PAL, CHS, CHI, F3H, FLS) and promotes flavonoid accumulation in species such as *Chrysanthemum morifolium* and wheat [55,56]. Together, these findings emphasize light and water availability as key ecological drivers shaping flavonoid profiles in Thymelaeaceae.

Our findings show that the flavonoid profiles of Korean Thymelaeaceae preserve clear chemotaxonomic signals despite ecological heterogeneity. Rather than simple co-occurrence, the diagnostic patterns of presence/absence and relative abundance among L7OG, yuankanin, luteolin, apigenin, hydroxygenkwanin, and genkwanin consistently differentiated lineages within Thymelaeaceae. *W. genkwa* and *W. ganpi* formed sister subclades within *Wikstroemia*, in line with recent DNA-based placements [9,10], indicating that environmental variability did not override the underlying chemical fingerprint. Within *Daphne*, *D. kiusiana* and *D. jejudoensis* clustered tightly, whereas *D. pseudomezereum* var. *koreana* remained distinct, again mirroring their reported morphological and genetic affinities [57,58,59]. Importantly, these chemotaxonomic patterns also show broad congruence with previously reported morphological traits (e.g., leaf arrangement, ovary structure) and molecular phylogenetic results, reinforcing that secondary metabolite profiles provide reliable diagnostic value when integrated with phenotypic and genetic evidence [5,6]. By contrast, *W. trichotoma* exhibited a restricted, apigenin-biased profile. While this yielded separation in HCA, the limited multi-compound signal (including near-LOD/LOQ non-detections) likely reduced statistical contrasts on some axes, and thus its placement should be interpreted cautiously. This pattern is consistent with previous reports showing that apigenin and its glycosides are dominant in *W. trichotoma* [60] although other abundant non-target metabolites such as diosmetin or flavanone derivatives were also identified. Hence, the apparent restriction may partly reflect the limited set of quantified markers rather than the true phytochemical diversity of this species. Comparative phytochemical reviews have emphasized that *Wikstroemia* taxa often accumulate methoxylated flavones such as hydroxygenkwanin and genkwanin, whereas *Daphne* taxa are generally characterized by luteolin- and apigenin-rich profiles [11,12]. Our results showed comparable tendencies, with *W. genkwa* and *W. ganpi* distinguished by relatively higher hydroxygenkwanin and genkwanin contents, while *Daphne* species tended to maintain more stable luteolin/apigenin patterns. These tendencies suggest that flavonoid constitution retain chemotaxonomic value for distinguishing lineages within Thymelaeaceae.

Flavonoid composition has repeatedly been shown to retain diagnostic value at taxonomic levels. *Epilobium* taxa displayed stable metabolic fingerprints across contrasting environments, underscoring their utility as chemotaxonomic markers rather than environmentally labile traits [61]. At a broader scale, comparative surveys across genera and families have demonstrated that characteristic flavonoid profiles consistently resolve taxonomic ambiguities, demonstrating their robustness as lineage-specific markers [62]. Consistent with this evidence, macroevolutionary analyses in *Helianthus* revealed that secondary metabolite profiles, including flavonoids, tracked phylogenetic relationships across the genus [63]. While providing useful insights, the present study also has areas that warrant careful consideration. Our clustering relied on a narrow set of six flavonoids and was limited to Korean species, meaning that extrapolations at the genus level should be treated with caution. Moreover, the presence of unassigned peaks in the chromatograms indicates that further metabolites remain to be identified. Therefore, future research should employ comprehensive metabolomics approaches, including UPLC-MS/MS and NMR, to capture the complete chemical profile, discover additional chemotaxonomic markers, and refine the assessment of similarity and distinctness among taxa.

In summary, this study demonstrates that integrating chemical and environmental perspectives can strengthen taxonomic frameworks and support both classification and chemotaxonomic evidence in Thymelaeaceae, thereby providing clearer insights into long-standing ambiguities between *Daphne* and *Wikstroemia*. This dual stability and plasticity highlight the value of these taxa as reliable chemical markers and reservoirs of phytochemical diversity. By incorporating ecological variability, future studies may achieve a more dynamic understanding of lineage boundaries and adaptive strategies. Such integrative perspectives also emphasize the importance of habitat heterogeneity in conservation and the sustainable utilization of phytochemicals in Thymelaeaceae.

## 4. Materials and Methods

### 4.1. Plant Samples

**Figure 5:**
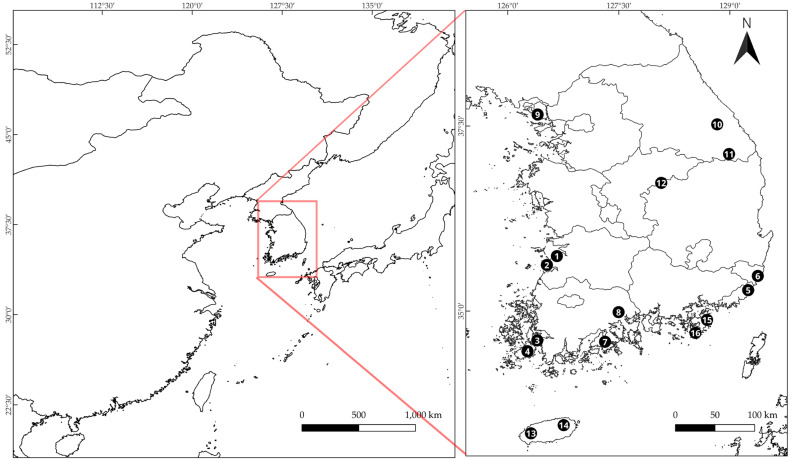
Sampling sites of Korean *Daphne* and *Wikstroemia* species. Black circles with numbers correspond to the population numbers (No.) in Table 1.

Leaf samples of six Thymelaeaceae species naturally distributed in South Korea were collected between April and June 2023. Mature leaves were collected to minimize developmental variation. In total, 70 samples representing 16 natural populations were obtained. Sampling localities were selected to represent the known distribution ranges of each species, and GPS coordinates were recorded for all sites (Table 1; Figure 5). Species identification followed the morphological keys and was confirmed by Hyun-Jun Kim (Forest Medicinal Resources Research Center, National Institute of Forest Science, Republic of Korea) [4,5]. Voucher information was documented as high-resolution photographs deposited in the digital archive of the Forest Medicinal Resources Research Center herbarium (FMRC).

Freshly collected leaves were immediately transported on ice to the laboratory and freeze-dried to a constant weight. The mean drying ratio (dry weight/fresh weight) was 29.7%. The dried samples were finely powdered using a mortar and pestle, and stored at −20 °C until extraction. For each extraction, 1.0 g of freeze-dried leaf powder was mixed with 10 mL of 99.9% methanol and sonicated for 60 min using an ultrasonic extractor (JAC-5020, KODO Co., Ltd., Hwaseong, Republic of Korea). The extracts were centrifuged at 3000 rpm for 5 min (UM-1238, Bio-Medical Science Co., Ltd., Seoul, Republic of Korea), and the supernatants were sequentially filtered through qualitative filter paper and a 0.2 μm membrane filter (Whatman, Maidstone, UK) before UPLC analysis.

### 4.2. Chemical Analysis

**Figure 6:**
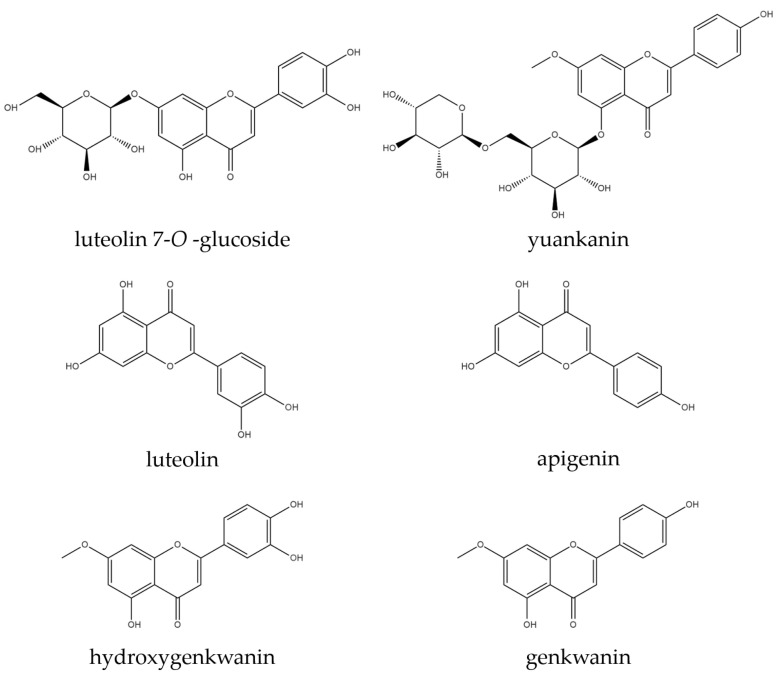
Chemical structures of six flavonoids.

Standards of luteolin-7-*O*-glucoside (L7OG), yuankanin, luteolin, apigenin, genkwanin, and hydroxygenkwanin (≥98%) were purchased from ChemFaces (Biochemical Co., Ltd., Wuhan, China) (Figure 6). HPLC-grade acetonitrile, methanol, and distilled water were obtained from J.T. Baker (Avantor, Inc., Radnor, PA, USA), and formic acid was purchased from Sigma-Aldrich (St. Louis, MO, USA).

**Figure 7:**
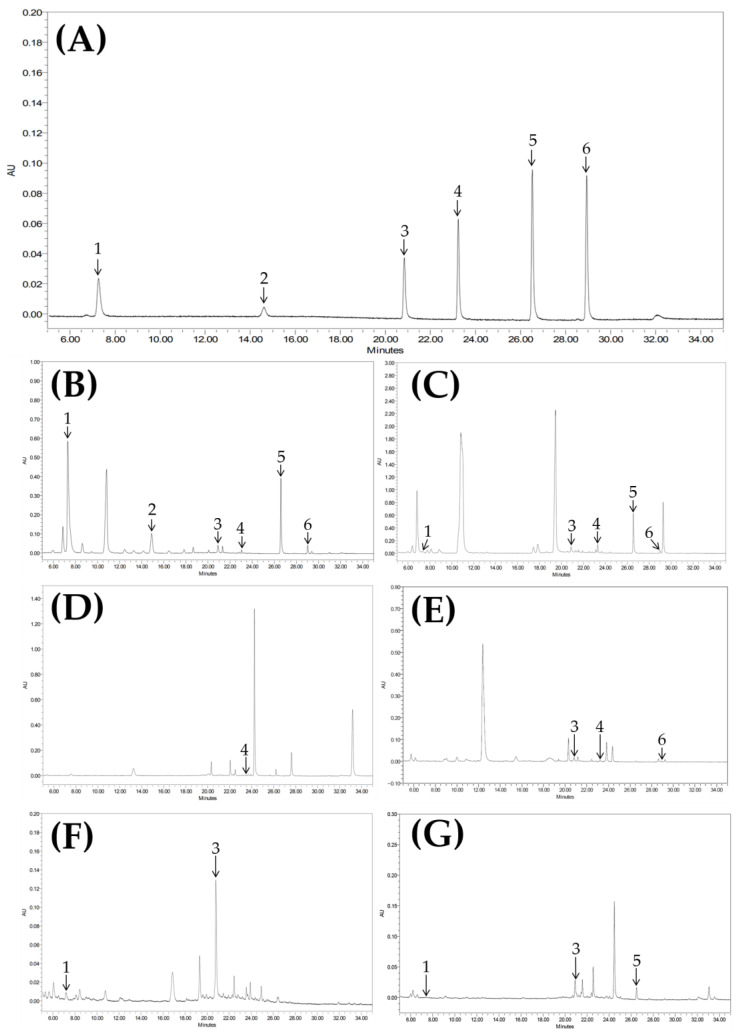
UPLC chromatogram of six flavonoids of the standard mixture (**A**) and *Wikstroemia genkwa* (**B**), *W. ganpi* (**C**), *W. trichotoma* (**D**), *Daphne pseudomezereum* var. *koreana* (**E**), *D. jejudoensis* (**F**), *D. kiusiana* (**G**), luteolin-7-*O*-glucoside (1), yuankanin (2), luteolin (3), apigenin (4), hydroxygenkwanin (5), genkwanin (6).

Chromatographic analyses for the simultaneous quantification of six flavonoids were performed using a Waters Alliance UPLC system equipped with a UV detector and an ACQUITY UPLC BEH C18 column (2.1 mm × 100 mm, 1.7 μm, 100Å; Waters Corp., Milford, MA, USA). The column oven and sample compartment were maintained at 30 °C and 10 °C, respectively. The mobile phase consisted of solvent A (0.1% formic acid in distilled water) and solvent B (0.1% formic acid in acetonitrile) under the following gradient: 0–15 min, 15% B; 15–23 min, 17% B; 23–30 min, 35% B; 30–35 min, 50% B; 35–35.1 min, 90% B; 35.1–38 min, 15% B; and 38.1–40 min, 15% B. Flow rate, injection volume, and detection wavelength were set at 0.2 mL/min, 1.0 μL, and 254 nm, respectively. Data acquisition and instrument control were managed using Empower 3 software (Waters Co., Milford, MA, USA), each extract was analyzed in triplicate using UPLC–UV, and mean values were calculated (Figure 7). The number of biological replicates per taxon is presented in Table 1.

Method validation was conducted in accordance with the International Council for Harmonization (ICH) guidelines to assess linearity, sensitivity, and precision. Calibration curves for each standard were constructed over the following concentration ranges: L7OG (1.0–400.0 μg/mL), yuankanin (25.0–400.0 μg/mL), luteolin (0.5–16.0 μg/mL), apigenin (1.0–32.0 μg/mL), hydroxygenkwanin (5.0–400.0 μg/mL), and genkwanin (5.0–160.0 μg/mL). Linearity was evaluated using six to seven concentration levels for each analyte. Limits of detection (LOD) and quantification (LOQ) were determined under the present chromatographic conditions at signal-to-noise ratios of 3.3 and 10, respectively. Precision was evaluated for repeatability (intra-day, *n* = 3) and intermediate precision (inter-day, successive three days), and expressed as the relative standard deviation (RSD, %).

### 4.3. Environmental Variables

For each sampling site, geographic coordinates (latitude and longitude) and elevation were recorded using a handheld GPS device (GPSMAP 64s, Garmin Ltd., Olathe, KS, USA). Slope was measured with a clinometer and compass (Suunto Tandem, Suunto Oy, Vantaa, Finland). Canopy cover was assessed by photographing the overhead vegetation using a digital camera (Nikon D80, Nikon Corp., Kyoto, Japan) equipped with a fisheye lens (4.5 mm F2.8 EX DC, SIGMA Corp., Kawasaki, Japan). Hemispherical photographs were analyzed with the Gap Light Analyzer (GLA) software, version 2.0, to calculate the canopy cover index (CCI) [64].

Nineteen bioclimatic variables (BIO1–BIO19) describing temperature and precipitation patterns were obtained from the WorldClim v2.1 database [65] at a spatial resolution of 30 arc-seconds (~1 km^2^). Variables were extracted for each sampling coordinate using R (version 4.2.3; RStudio, Posit PBC, Boston, MA, USA).

### 4.4. Statistical and Multivariate Analysis

All statistical analyses were performed using R version 4.2.3. Flavonoid data were log10-transformed and mean-centered prior to analysis. Pearson correlation coefficients were calculated to assess relationships between flavonoid contents and environmental variables, and correlation matrices were visualized with the packages corrplot(v.0.95) and ggcorrplot2 (v. 3.5.2). Hierarchical cluster analysis (Euclidean distance, Ward’s method) and principal component analysis (PCA) were applied to standardized data to evaluate group separation and taxonomic relationships. Statistical significance was set at *p* < 0.05.

## 5. Conclusions

This study examined six Korean taxa of the family Thymelaeaceae (*W. genkwa*, *W. ganpi*, *W. trichotoma*, *D. pseudomezereum* var. *koreana*, *D. jejudoensis*, and *D. kiusiana*) by analyzing flavonoid profiles in relation to environmental variables. Flavonoid contents showed significant correlations with altitude, temperature, and canopy cover, yet hierarchical clustering and PCA consistently separated *Wikstroemia* and *Daphne* into two major clades with five subclades. Notably, *W. trichotoma* formed a distinct cluster characterized by its restricted flavonoid profile, while *D. kiusiana* and *D. jejudoensis* clustered closely together, and *D. pseudomezereum* var. *koreana* maintained a separate grouping. These results suggest that, although secondary metabolites exhibit environmental plasticity, overall, flavonoid patterns retain taxonomic indicators at both the generic and species levels.

## Figures and Tables

**Table 1:** Taxa, sample codes, collection coordinates and topographic information of Korean Thymelaeaceae populations.

Taxa	No	Abbr.	Sample No.	Lat.	Long.	Alt. (m)	Slope (%)	CCI (%)
Genus *Wikstroemia*								
*W. genkwa* (Siebold & Zucc.) Domke	1	WGEN	1-1 to 5	35.60	126.49	174.60	35.00	0.02
	2	WGEN	2-1 to 3	35.59	126.50	33.20	25.00	0.00
	3	WGEN	3-1 to 4	34.57	126.32	48.80	5.00	0.08
	4	WGEN	4-1	34.56	126.30	43.30	3.00	0.04
*W. ganpi* (Siebold & Zucc.) Maxim	5	WGAN	1-1 to 3	35.23	129.24	42.50	15.00	0.00
	6	WGAN	2-1 to 6	35.54	129.41	268.00	45.00	0.81
	7	WGAN	3-1 to 6	34.60	127.42	73.00	60.00	0.08
*W. trichotoma* (Thunb.) Makino	8	WTRI	1-1 to 6	34.98	127.47	78.00	40.00	0.33
	9	WTRI	2-1 to 6	37.72	126.38	123.60	14.00	0.59
Genus *Daphne*								
*D. pseudomezereum* var. *koreana* (Nakai) Hamaya	10	DPSE	1-1 to 7	37.59	128.89	956.00	30.00	0.54
	11	DPSE	2-1	36.73	128.02	1192.00	43.00	0.66
	12	DPSE	3-1 to 3	37.15	128.89	756.00	25.00	0.59
*D. jejudoensis* M.Kim	13	DJEJ	1-1 to 11	33.54	126.72	190.80	10.00	0.76
	14	DJEJ	2-1 to 3	33.32	126.29	87.60	15.00	0.69
*D. kiusiana* Miq.	15	DKIU	1-1 to 3	34.73	128.61	115.00	8.00	0.85
	16	DKIU	2-1 to 2	34.73	128.68	107.00	20.00	0.88

Abbr., abbreviation; Lat., latitude; Long., longitude; Alt., altitude; CCI, canopy cover index

**Table 2:** Linear regression, LOD and LOQ of six flavonoids.

Compound	Regression Equation	Correlation Coefficient	Linear Range (μg/mL)	LOD (μg/mL)	LOQ (μg/mL)
L7OG	y ^a^ = 5115.6x ^b^ + 3158.9	0.9999	1.0–400.0	0.05	0.15
yuankanin	y = 1950.1x + 2021.7	0.9999	25.0–400.0	0.06	0.19
luteolin	y = 70,466x + 119.01	1.0000	0.5–16.0	0.01	0.02
apigenin	y = 10,596x − 2562.8	0.9996	1.0–32.0	0.04	0.11
hydroxygenkwanin	y = 15,859x − 8070.6	1.0000	5.0–400.0	0.06	0.17
genkwanin	y = 14,910x + 7178.8	0.9998	5.0–160.0	0.05	0.15

^a^ y: peak area; ^b^ x: concentration of compound (µg/mL).

**Table 3:** Intra-, Inter-day precision for identifying the six flavonoids.

Compound	Concentration (µg/mL)	Intra-Day ^a^		Inter-Day ^b^	
		Concentration Found (µg/mL)	RSD (%)	Concentration Found (µg/mL)	RSD (%)
luteolin 7-*O*-glucoside	10	9.40	0.07	9.42	0.21
	20	19.91	0.05	19.96	0.27
	40	41.55	0.55	41.75	0.08
yuankanin	25	24.44	0.44	24.46	0.09
	50	49.01	0.24	49.15	0.31
	100	100.16	0.06	100.23	0.06
luteolin	1	1.00	0.56	0.99	0.23
	4	4.11	0.03	4.10	0.22
	16	16.18	0.04	16.18	0.01
apigenin	4	4.06	0.09	4.05	0.39
	8	8.20	0.05	8.19	0.12
	16	16.09	0.22	16.15	0.29
hydroxygenkwanin	5	5.16	0.55	5.16	0.36
	10	10.04	0.08	10.03	0.09
	40	40.04	0.02	40.06	0.04
genkwanin	5	4.94	0.20	4.93	0.30
	10	10.04	0.01	10.03	0.10
	40	40.81	0.04	40.84	0.14

^a^ Sample analyzed three times on one day; ^b^ Sample analyzed each day on 3 consecutive days with three injections per day. RSD, Relative Standard Deviation.

## Data Availability

The data presented in this study are available under permission from the corresponding author upon reasonable request.

## Supplementary Materials

The following supporting information can be downloaded at: https://www.mdpi.com/article/10.3390/plants14193059/s1, Table S1: Environmental variables for each sampling population; Table S2: Concentration of six flavonoids in leaf extracts of Korean Thymelaeaceae taxa; Table S3: Pearson’s correlation coefficients between flavonoids and environmental variables; Table S4: Pearson’s correlation coefficients between flavonoids and bioclimatic variables (BIO1–BIO19).
