# Supplementary material for: Chemotaxonomic Insights into Korean Daphne spp. and Wikstroemia spp. by Integrating Flavonoid Contents with Ecological Factors
Source: Plants (Basel). 2025 Oct 3;14(19):3059. doi: 10.3390/plants14193059 (PMC12526056; doi:10.3390/plants14193059)
Supplement: Supplementary file 1 [file plants-14-03059-s001.zip › plants-3884668-supplementary.pdf]

Supplementary Data

Chemotaxonomic Insights into Korean *Daphne* spp. and *Wikstroemia* spp. by Integrating Flavonoid contents with Ecological Factors

Yonghwan Son [1], Ji Ah Kim[1], Ho Jun Son[1], Hyun-Jun Kim[1,*], and Wan-Geun Park[2,*]

[1] Forest Medicinal Resources Research Center, National Institute of Forest Science, Yeongju-si 36040, Republic of Korea; thsdydghks@korea.kr (Y.S.); jiahkim@korea.kr (J.A.K.); shjj7740@korea.kr (H.J.S.)
[2] Departments of Forest Resources, College of Forest and Environmental Sciences, Kangwon National University,
Chuncheon 24341, Republic of Korea; wgpark@kangwon.ac.kr (W.-G.P.)
* Correspondence: mind4938@korea.kr

Table S1. Environmental variables for each sampling population

Point	LAT	LONG	Bio1	Bio2	Bio3	Bio4	Bio5	Bio6	Bio7	Bio8	Bio9	Bio10	Bio11	Bio12	Bio13	Bio14	Bio15	Bio16	Bio17	Bio18	Bio19
WGEN 1	35.60	126.49	12.60	9.07	27.15	911.25	29.20	-4.20	33.40	23.38	1.25	23.53	1.25	1184.00	245.00	34.00	73.13	614.00	107.00	598.00	107.00
WGEN 2	35.59	126.50	12.77	9.07	27.15	911.68	29.40	-4.00	33.40	23.58	1.43	23.72	1.43	1170.00	241.00	33.00	72.91	606.00	106.00	590.00	106.00
WGEN 3	34.57	126.32	14.06	8.20	26.62	843.66	29.40	-1.40	30.80	23.92	3.58	24.30	3.58	1222.00	223.00	30.00	64.77	597.00	115.00	549.00	115.00
WGEN 4	34.56	126.30	14.00	8.14	26.43	844.91	29.30	-1.50	30.80	23.88	3.50	24.23	3.50	1227.00	224.00	30.00	64.65	599.00	116.00	549.00	116.00
WGAN 1	35.23	129.24	14.19	8.00	27.12	780.20	28.60	-0.90	29.50	23.28	4.48	23.72	4.48	1350.00	234.00	26.00	65.17	646.00	110.00	610.00	110.00
WGAN 2	35.54	129.41	13.34	8.66	28.11	813.78	28.30	-2.50	30.80	22.92	3.13	23.13	3.13	1278.00	232.00	27.00	65.43	618.00	111.00	591.00	111.00
WGAN 3	34.60	127.42	13.56	8.50	27.07	854.44	28.90	-2.50	31.40	23.60	2.80	23.78	2.80	1474.00	271.00	25.00	72.31	756.00	102.00	687.00	102.00
WTRI 1	34.98	127.47	12.93	9.24	27.92	896.80	29.00	-4.10	33.10	23.58	1.60	23.58	1.60	1405.00	287.00	24.00	77.32	754.00	94.00	754.00	94.00
WTRI 2	37.72	126.38	10.65	8.16	22.85	1014.76	27.40	-8.30	35.70	22.30	-2.43	22.52	-2.43	1260.00	322.00	18.00	98.59	770.00	60.00	744.00	60.00
DPSE 1	37.59	128.89	7.90	9.81	28.02	942.10	24.40	-10.60	35.00	18.62	-4.12	19.12	-4.12	1361.00	274.00	37.00	73.55	718.00	126.00	682.00	126.00
DPSE 2	36.73	128.02	8.43	10.55	28.75	995.30	25.70	-11.00	36.70	20.35	-4.30	20.35	-4.30	1371.00	342.00	25.00	88.53	780.00	89.00	780.00	89.00
DPSE 3	37.15	128.89	6.82	10.65	28.55	990.08	24.20	-13.10	37.30	17.98	-5.93	18.58	-5.93	1393.00	287.00	38.00	73.47	721.00	131.00	712.00	131.00
DJEJ 1	33.54	126.72	14.82	6.78	24.49	770.48	28.80	1.10	27.70	23.92	7.58	24.45	5.42	1771.00	278.00	53.00	54.94	792.00	201.00	728.00	204.00
DJEJ 2	33.32	126.29	14.69	6.78	24.85	752.34	28.50	1.20	27.30	23.55	7.77	24.08	5.55	1768.00	284.00	51.00	56.13	791.00	193.00	700.00	195.00
DKIU 1	34.73	128.61	13.76	8.13	26.99	785.17	28.30	-1.80	30.10	22.80	3.90	23.37	3.90	1682.00	302.00	29.00	67.89	818.00	127.00	753.00	127.00
DKIU 2	34.73	128.68	14.15	7.61	26.98	739.28	28.10	-0.10	28.20	22.72	5.08	23.33	5.08	1674.00	300.00	29.00	67.53	811.00	125.00	748.00	125.00

LAT, latitude; Long, longitude

Table S2. Concentration of six flavonoids in leaf extracts of Korean Thymelaeaceae taxa

TAXA.	Concentration (μg /mL)					
	**luteolin 7-*O*-glucoside**	**yuankanin**	**luteolin**	**apigenin**	**hydroxy genkwanin**	**genkwanin**
**Genus *Wikstroemia***						
WGEN 1-1	1336.36 ± 11.64	640.02 ± 10.37	3.05 ± 0.06	1.21 ± 0.04	142.48 ± 1.9	15.08 ± 0.32
WGEN 1-2	532.89 ± 3.67	517.49 ± 1.83	1.41 ± 0.03	ND[a]	118.21 ± 0.58	7.99 ± 0.14
WGEN 1-3	1938.68 ± 6.63	435.58 ± 4.31	15.26 ± 0.12	2.77 ± 0.1	228.59 ± 1.2	15.33 ± 0.23
WGEN 1-4	1509.12 ± 10.75	668.48 ± 0.57	3.18 ± 0.04	1.34 ± 0.06	121.35 ± 0.54	14.39 ± 0.24
WGEN 1-5	1277.74 ± 5.77	815.78 ± 8.12	7.84 ± 0.05	2.68 ± 0.1	325.76 ± 0.3	44.11 ± 0.14
WGEN 2-1	1483.6 ± 3.07	538.21 ± 11.06	7.12 ± 0.05	5.77 ± 0.04	542.67 ± 2.86	65.87 ± 0.43
WGEN 2-2	730.97 ± 0.8	138.39 ± 1.92	4.93 ± 0.09	2.83 ± 0.09	815.66 ± 5.03	45.36 ± 0.23
WGEN 2-3	945.34 ± 5.36	158.73 ± 4.26	8.42 ± 0.09	2.87 ± 0.09	1177.68 ± 9.36	75.8 ± 0.61
WGEN 3-1	19.35 ± 0.29	722.76 ± 6.8	0.28 ± 0	0.61 ± 0.02	21.05 ± 0.07	1.32 ± 0.07
WGEN 3-2	59.29 ± 0.3	1023.14 ± 24.84	0.31 ± 0	ND	27.45 ± 0.77	1.14 ± 0.09
WGEN 3-3	40.17 ± 0.65	814.71 ± 12.3	0.39 ± 0.01	1.41 ± 0.04	32.19 ± 0.46	2.35 ± 0.05
WGEN 3-4	8.72 ± 0.11	682.97 ± 8.99	0.16 ± 0	ND	15.15 ± 0.21	1.61 ± 0.04
WGEN 4-1	87.95 ± 0.3	158.06 ± 1.89	1.74 ± 0.03	1.08 ± 0.06	897.03 ± 1.04	79.9 ± 0.34
WGAN 1-1	9.99 ± 1.33	ND	ND	87.29 ± 15.1	482.33 ± 77.92	17.78 ± 2.91
WGAN 1-2	56.7 ± 0.44	ND	28.69 ± 0.24	12.68 ± 0.61	26.07 ± 0.21	26.68 ± 0.35
WGAN 1-3	6.37 ± 0.4	ND	8.57 ± 0.6	8.84 ± 0.91	188.58 ± 17.31	7.64 ± 0.64
WGAN 2-1	1.29 ± 0.2	ND	2.24 ± 0.21	1.19 ± 0.17	158.93 ± 16.66	2.53 ± 0.32
WGAN 2-2	8.13 ± 0.87	ND	9.87 ± 1	21.49 ± 1.85	363.47 ± 34.81	11.07 ± 1.34
WGAN 2-3	8.21 ± 0.45	ND	16.83 ± 0.2	8.54 ± 0.1	1260.06 ± 4.71	40.39 ± 1.01
WGAN 2-4	19.7 ± 0.92	ND	33.5 ± 0.34	38.37 ± 0.78	1027.49 ± 6.11	25.42 ± 0.58
WGAN 2-5	10.03 ± 0.21	ND	27.92 ± 0.15	35.17 ± 0.79	975.28 ± 6.83	37.02 ± 0.2
WGAN 2-6	22.65 ± 0.29	ND	27.2 ± 0.16	10.17 ± 0.41	1339.61 ± 14.92	45.92 ± 0.55
WGAN 3-1	12.66 ± 0.04	ND	1.63 ± 0.02	14.42 ± 0.14	251.9 ± 1.33	13.8 ± 0.21
WGAN 3-2	24.46 ± 0.14	ND	7.37 ± 0.18	47.72 ± 0.47	217.21 ± 0.83	17.15 ± 0.65
WGAN 3-3	10.78 ± 0.21	ND	0.77 ± 0	45.06 ± 0.65	46.68 ± 0.72	1.46 ± 0.05
WGAN 3-4	19.68 ± 0.67	ND	2.92 ± 0.07	39.25 ± 0.75	84.24 ± 0.72	4.34 ± 0.05
WGAN 3-5	28.21 ± 0.16	ND	2.47 ± 0.01	46.35 ± 0.77	50.46 ± 0.36	3.62 ± 0.03
WGAN 3-6	10.27 ± 0.22	ND	1.17 ± 0.01	72.53 ± 0.45	18.48 ± 0.11	0.87 ± 0.03
WTRI 1-1	ND	ND	ND	3.72 ± 0.07	ND	ND
WTRI 1-2	1.88 ± 0.02	ND	ND	4.1 ± 0.06	ND	ND
WTRI 1-3	ND	ND	ND	3.31 ± 0.08	ND	ND
WTRI 1-4	1.83 ± 0.06	ND	ND	5.28 ± 0.02	ND	ND
WTRI 1-5	ND	ND	ND	3.43 ± 0.04	ND	ND
WTRI 1-6	ND	ND	ND	3.97 ± 0.08	ND	ND
WTRI 2-1	ND	ND	ND	4.1 ± 0.01	ND	ND
WTRI 2-2	ND	ND	ND	2.84 ± 0.08	ND	ND
WTRI 2-3	ND	ND	ND	2.34 ± 0.02	ND	ND
WTRI 2-4	ND	ND	ND	4.2 ± 0.01	ND	ND
WTRI 2-5	ND	ND	ND	2.71 ± 0.02	ND	ND
WTRI 2-6	ND	ND	ND	2.87 ± 0.07	ND	ND
**Genus *Daphne***						
DPSE 1-1	ND	ND	9.41 ± 0.33	9.67 ± 0.28	ND	0.83 ± 0.02
DPSE 1-2	ND	ND	8.66 ± 0.03	2.92 ± 0.03	ND	0.89 ± 0.02
DPSE 1-3	ND	ND	7.04 ± 0.01	4.07 ± 0.04	ND	0.78 ± 0.01
DPSE 1-4	ND	ND	5.72 ± 0.13	1.93 ± 0.06	ND	0.86 ± 0.05

DPSE 1-5	ND	ND	8.85 ± 0.04	1.71 ± 0.04	ND	0.5 ± 0.03
DPSE 1-6	ND	ND	8.19 ± 0.04	1.97 ± 0.03	ND	0.1 ± 0.01
DPSE 1-7	ND	ND	5.9 ± 0.06	2.79 ± 0.07	ND	0.69 ± 0.04
DPSE 2-1	10.77 ± 0.16	ND	2.51 ± 0.01	2.59 ± 0.04	ND	0.43 ± 0.02
DPSE 3-1	ND	ND	3.5 ± 0.03	1.58 ± 0.03	ND	ND
DPSE 3-2	6.78 ± 0.09	ND	3.82 ± 0.06	2.59 ± 0.08	ND	1.25 ± 0.04
DPSE 3-3	ND	ND	2.07 ± 0	2.33 ± 0.06	ND	1.27 ± 0.01
DJEJ 1-1	4.5 ± 0.12	ND	6.63 ± 0.06	ND	ND	ND
DJEJ 1-2	13.46 ± 0.09	ND	12.46 ± 0.11	ND	ND	ND
DJEJ 1-3	10.05 ± 0.18	ND	11.8 ± 0.07	ND	ND	ND
DJEJ 1-4	ND	ND	4.68 ± 0.04	ND	ND	ND
DJEJ 1-5	ND	ND	8.26 ± 0.08	ND	ND	ND
DJEJ 1-6	ND	ND	5.31 ± 0.03	ND	ND	ND
DJEJ 1-7	14.06 ± 0.09	ND	8.27 ± 0.02	ND	ND	ND
DJEJ 1-8	ND	ND	1.81 ± 0.02	ND	ND	ND
DJEJ 1-9	10.26 ± 0.1	ND	3.94 ± 0	ND	ND	ND
DJEJ 1-10	9.22 ± 0.05	ND	4.77 ± 0.11	ND	ND	ND
DJEJ 1-11	ND	ND	2.03 ± 0.03	ND	ND	ND
DJEJ 2-1	9.27 ± 0.03	ND	9.57 ± 0.04	ND	ND	ND
DJEJ 2-2	10.22 ± 0.05	ND	9.27 ± 0.03	ND	ND	ND
DJEJ 2-3	ND	ND	3.69 ± 0	ND	ND	ND
DKIU 1-1	25.26 ± 0.49	ND	4.93 ± 0.14	ND	1.41 ± 0.07	ND
DKIU 1-1	17.95 ± 0.37	ND	4.91 ± 0.03	ND	1.85 ± 0.04	ND
DKIU 1-3	66.37 ± 0.24	ND	21.75 ± 0.13	ND	4.76 ± 0.08	ND
DKIU 2-1	21.31 ± 0.2	ND	15.15 ± 0.11	ND	1.39 ± 0.08	ND
DKIU 2-2	15.96 ± 0.31	ND	7.23 ± 0.03	ND	1.47 ± 0.02	ND

[a]ND: not detected

Table S3. Pearson's correlation coefficients between flavonoids and environmental variables

	Correlation coefficient (*r*)[a]				
	**Latitude**	**Longitude**	**Altitude**	**Slope**	**CCI**
**L7OG**	0.049	-0.311**	-0.150	0.111	-0.488**
	(0.686)	(0.009)	(0.215)	(0.358)	(0.000)
**yuankanin**	-0.068	-0.421**	-0.215	-0.158	-0.558**
	(0.578)	(0.000)	(0.074)	(0.192)	(0.000)
**luteolin**	-0.066	0.482**	0.084	0.090	0.295*
	(0.590)	(0.000)	(0.490)	(0.460)	(0.013)
**apigenin**	-0.046	0.267*	-0.156	0.527**	-0.318**
	(0.705)	(0.025)	(0.197)	(0.000)	(0.007)
**hydroxygenkwanin**	0.015	0.220	-0.148	0.253*	-0.106
	(0.904)	(0.067)	(0.222)	(0.034)	(0.382)
**genkwanin**	0.007	-0.007	-0.203	0.128	-0.369**
	(0.954)	(0.951)	(0.092)	(0.292)	(0.002)
**Total**	0.010	-0.204	-0.227	0.137	-0.516**
	(0.936)	(0.091)	(0.058)	(0.257)	(0.000)

L7OG, luteolin-7-*O*-glucoside; CCI, canopy cover index.

[a]Positive and negative values indicate the direction of correlation, with p-values shown in brackets (**$p < 0.01$, *$p < 0.05$).

Table S4. Pearson's correlation coefficients between flavonoids and bioclimatic variables (BIO1–BIO19)

	Correlation coefficient (*r*)[a]						
	**L7OG**	**yuankanin**	**luteolin**	**apigenin**	**hydroxy genkwanin**	**genkwanin**	**Total**
Bio1	0.030 (0.807)	0.134 (0.268)	0.124 (0.308)	0.127 (0.296)	0.146 (0.227)	0.131 (0.280)	0.133 (0.272)
Bio2	0.191 (0.112)	0.089 (0.464)	-0.071 (0.561)	0.058 (0.631)	0.112 (0.356)	0.134 (0.269)	0.189 (0.117)
Bio3	0.128 (0.292)	0.094 (0.441)	0.230 (0.056)	0.211 (0.080)	0.282* (0.018)	0.230 (0.056)	0.239* (0.046)
Bio4	0.179 (0.137)	0.082 (0.500)	-0.356** (0.002)	-0.097 (0.426)	-0.090 (0.459)	-0.001 (0.992)	0.081 (0.506)
Bio5	0.272* (0.023)	0.338** (0.004)	-0.025 (0.839)	0.131 (0.281)	0.224 (0.062)	0.278* (0.020)	0.371** (0.002)
Bio6	-0.020 (0.871)	0.087 (0.471)	0.176 (0.145)	0.088 (0.471)	0.108 (0.375)	0.084 (0.491)	0.072 (0.556)
Bio7	0.176 (0.144)	0.063 (0.607)	-0.260* (0.030)	-0.051 (0.673)	-0.028 (0.817)	0.035 (0.775)	0.103 (0.396)
Bio8	0.164 (0.175)	0.240* (0.046)	-0.026 (0.832)	0.123 (0.309)	0.168 (0.165)	0.197 (0.103)	0.252* (0.035)
Bio9	-0.060 (0.622)	0.022 (0.856)	0.183 (0.130)	0.043 (0.723)	0.064 (0.601)	0.030 (0.802)	0.060 (0.961)
Bio10	0.132 (0.277)	0.227 (0.059)	-0.006 (0.958)	0.101 (0.406)	0.139 (0.250)	0.168 (0.163)	0.216 (0.072)
Bio11	-0.025 (0.835)	0.079 (0.518)	0.205 (0.089)	0.112 (0.357)	0.133 (0.272)	0.098 (0.421)	0.078 (0.521)
Bio12	-0.413** (0.000)	-0.474** (0.000)	0.051 (0.672)	-0.114 (0.347)	-0.403** (0.001)	-0.453** (0.000)	-0.577** (0.000)
Bio13	-0.330** (0.005)	-0.534** (0.000)	-0.311** (0.009)	-0.218 (0.070)	-0.556** (0.000)	-0.553** (0.000)	-0.628** (0.000)
Bio14	0.002 (0.988)	-0.054 (0.655)	0.102 (0.402)	-0.326** (0.006)	-0.190 (0.115)	-0.150 (0.215)	-0.111 (0.359)
Bio15	0.072 (0.552)	-0.040 (0.743)	-0.320** (0.007)	0.023 (0.853)	-0.089 (0.465)	-0.046 (0.707)	-0.016 (0.893)
Bio16	-0.489** (0.000)	-0.637** (0.000)	-0.202 (0.093)	-0.115 (0.344)	-0.601** (0.000)	-0.629** (0.000)	-0.768** (0.000)
Bio17	-0.151 (0.211)	-0.147 (0.224)	0.162 (0.181)	-0.240* (0.045)	-0.181 (0.134)	-0.193 (0.110)	-0.221 (0.066)
Bio18	-0.426** (0.000)	-0.648** (0.000)	-0.258* (0.031)	-0.174 (0.151)	-0.587** (0.000)	-0.613** (0.000)	-0.732** (0.000)
Bio19	-0.152 (0.208)	-0.149 (0.217)	0.158 (0.191)	-0.241* (0.045)	-0.183 (0.130)	-0.195 (0.106)	-0.223 (0.063)

L7OG, luteolin-7-*O*-glucoside.

[a]Positive and negative values indicate the direction of correlation, with p-values shown in brackets (**$p < 0.01$, *$p < 0.05$).
